# Comparison of the Analgesic Efficacy between Levobupivacaine 0.25% and Ropivacaine 0.375% for PENG (Pericapsular Nerve Group) Block in the Context of Hip Fracture Surgery of Elderly Patients: A Single-Center, Randomized, and Controlled Clinical Trial

**DOI:** 10.3390/jcm13030770

**Published:** 2024-01-29

**Authors:** Daniel Salgado-García, Agustín Díaz-Álvarez, José Luis González-Rodríguez, María Rocío López-Iglesias, Eduardo Sánchez-López, Manuel Jesús Sánchez-Ledesma, María Isabel Martínez-Trufero

**Affiliations:** 1 Department of Anesthesiology and Critical Care, Salamanca University Hospital, Gerencia Regional de Salud de Castilla y León (SACYL), Paseo de la Transición Española, 37007 Salamanca, Castilla y León, Spain; danielsalgadomd@gmail.com (D.S.-G.); jlgonzalezr@saludcastillayleon.es (J.L.G.-R.); mrlopezig@saludcastillayleon.es (M.R.L.-I.); esanchezl@saludcastillayleon.es (E.S.-L.); mjsanchezl@saludcastillayleon.es (M.J.S.-L.); imtrufero@saludcastillayleon.es (M.I.M.-T.); 2 Department of Surgery, Faculty of Medicine, Salamanca University, Calle Alfonso X el Sabio, 37007 Salamanca, Castilla y León, Spain; 3 Instituto de Investigación Biomédica de Salamanca de la FIESCYL (IBSAL-FIESCYL), Paseo de San Vicente 182, 37007 Salamanca, Castilla y León, Spain

**Keywords:** frail elderly, hip fractures, levobupivacaine, nerve block, pain management, pain measurement, pain postoperative, ropivacaine, ultrasonography

## Abstract

Previous studies have compared levobupivacaine versus ropivacaine in various peripheral nerve blocks in terms of block duration, quality of analgesia, and onset time, but this has not occurred in the PENG block. Here, a single-center, randomized, and controlled clinical trial is presented. One hundred and twenty patients older than 65 years suffering from hip fractures and surgically treated at our institution under spinal anesthesia were eligible for participation; of them, one hundred and eight were analyzed. Patients were randomized to receive ultrasound-guided PENG blocks using 20 mL of either 0.25% levobupivacaine or 0.375% ropivacaine (both of which are equipotent concentrations). The primary endpoint was to compare the analgesic duration (time to first rescue) and analgesic quality (pain scores using the VAS, PAINAD, and AlgoPlus scales) between the groups. Secondary endpoints included comparing the onset time, describing the need for and type of rescue analgesics, and possible associated adverse effects. There were no statistically significant differences in analgesic duration between levobupivacaine (median 861.0, IQR 960) and ropivacaine (median 1205.0, IQR 1379; *p* = 0.069). Likewise, the quality of analgesia and onset time were comparable among the groups. A small number of patients required opioids as rescue analgesics (4.6%). The possible associated adverse effects included postoperative infection (11.1%) and delirium (2.8%).

## 1. Introduction

Hip fracture is one of the most frequent surgical diagnoses in our environment, affecting a very aged and polypathological population, generally polymedicated and in which the presence of neurodegenerative diseases is not uncommon, all of which determines a high degree of frailty among these patients [1,2].

In this context, the debate on the most appropriate anesthetic technique for hip fracture surgery in the elderly is still ongoing between general and neuraxial anesthesia, as several studies have concluded the non-existence of differences between them regarding primary outcomes such as mortality, ambulation capacity, or the incidence of delirium [3,4,5]. However, other studies have determined that neuroaxial anesthesia is superior to general anesthesia in specific aspects, such as hospital stay or the development of postoperative complications [6,7,8]. Such is the case that these benefits have tilted the preference towards the use of spinal anesthesia in routine clinical practice, particularly in elderly patients [9].

Pain control in these patients represents an unquestionable added value, since it facilitates postsurgical recovery and early mobilization and reduces the appearance of complications and hospitalization time after surgery [9,10]. Furthermore, elderly patients are especially sensitive to opioid-related adverse effects [11,12,13].

The use of peripheral nerve blocks has been shown to effectively reduce pain and opioid consumption in the perioperative period of hip fractures. In addition, they improve clinical outcomes after surgery [14,15,16]. This has led to the inclusion of the femoral nerve block (FNB) and fascia iliaca compartment block (FICB) in two recent clinical guidelines for the management of hip fractures [17].

In 2018, based on new anatomical findings on the innervation of the hip [18], it was described the performance of the PENG (Pericapsular Nerve Group) block. It specifically blocks the nerve endings of the femoral and accessory obturator nerves responsible for innervating the anterior capsule of the hip, which is the most densely innervated region of the hip, theoretically without causing motor blockade of the quadriceps, as these nerves are purely sensory [19]. This block has proven to be effective in postoperative pain control [20], so it has become popular in hip fracture surgery, motivating studies to compare its efficacy with respect to FNB and FICB, apparently offering advantages in analgesic control with a lower degree of motor block [21,22].

Many studies can be found that try to define the most adequate local anesthetic between levobupivacaine and ropivacaine for different peripheral nerve blocks [23]. Given the novelty of the PENG block, to the best of our knowledge, there is no scientific evidence of which is the most adequate for this technique.

The aim of this study is to compare the analgesic duration and quality between equipotent doses of levobupivacaine and ropivacaine when used to perform PENG block in the context of hip fracture surgery in elderly patients.

## 2. Materials and Methods

### 2.1. Study Design and Ethics

A phase IV, single-center, randomized, and controlled clinical trial is proposed. There was no change to the protocol after the start of the trial. The study adheres to the Declaration of Helsinki, and particularly to the ethics of frail orthogeriatric patients. Its protocol obtained approval for implementation by the Ethics Committee for Drug Research of the Health Area of Salamanca (CEIm 20/1605 dated 5 October 2020) in accordance with the International Council for Harmonisation (ICH) guidelines for Good Clinical Practice (GCP). This study was registered and authorized prior to the recruitment of the first patient in the national clinical trials registry of the Spanish Agency of Medicines and Medical Devices (2020-004697-21 dated 11 December 2020). In addition, this study was registered in ClinicalTrials.gov (NCT04773301) and in EudraCT (2020-004697-21). The study adhered to the CONSORT guidelines for reporting clinical trials.

### 2.2. Patient Screening and Enrolment

Before starting any procedure related to the study, written informed consent was obtained from all patients according to the ICH and GCP guidelines. In the case of limitations in this regard, as in the case of patients with cognitive impairment, consent was obtained from a relative or legal representative. To ensure proper accuracy in the assessment of these patients, the use of the PAINAD and AlgoPlus scales was incorporated, both specifically designed for pain assessment in patients with cognitive impairment.

The inclusion criteria for this study comprised patients aged over 65 with hip fractures who were surgically treated at our institution. The exclusion criteria were refusal of the technique, allergies to any of the medications, coagulation disorders, local infection at the puncture site, or the presence of a femoral vascular prosthesis. The withdrawal criteria from the clinical trial consisted of withdrawal of consent; complications during surgery that, in the investigator’s opinion, could invalidate the study’s results (i.e., the need for conversion to general anesthesia, as we decided to conduct the study under spinal anesthesia); or inability to assess pain using the scales employed. The entire development took place at the Salamanca University Hospital by the authors of this publication, all of them medical specialists from the Anesthesiology and Intensive Care Service.

### 2.3. Intervention

Single-shot PENG and Femoral Lateral Cutaneous Nerve (FLCN) blocks were performed on the recruited patients whether with levobupivacaine or ropivacaine depending on the group. FLCN block is performed at our institution as part of multimodal analgesia to cover incisional pain, which is different from the pain originating from the hip fracture and its fixation that PENG block covers, thus having no influence on the target variables of this study.

**Figure 1:**
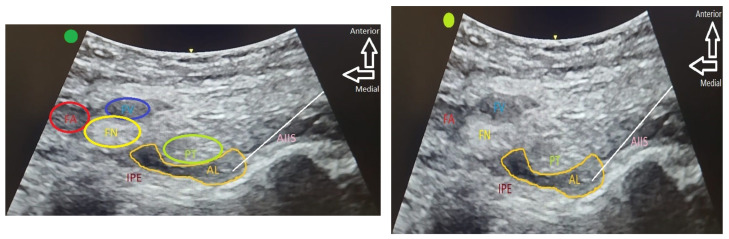
Details of the ultrasonographic implementation of the PENG block. In the left image, ultrasound exploration of the area where the block is performed is depicted, showcasing the most relevant anatomical structures of the region: the femoral artery (FA), femoral vein (FV), femoral nerve (FN), bony landmarks of the anterior inferior iliac spine (AIIS), and the iliopubic eminence (IPE), as well as the psoas tendon (PT). The latter three structures delineate the plane through which the branches of the femoral nerve and accessory obturator nerve traverse, contributing sensory innervation to the anterior hip capsule. Consequently, they define the area where the local anesthetic should be administered. The right image illustrates the distribution of the injected local anesthetic between the pectineus muscle and the psoas tendon.

These blocks were performed in the operating room at the patient’s bedside, prior to their transference to the operating table and after EKG, NIBP, and pulse oximetry monitoring, using a sterile technique and guided by ultrasound using a low-frequency convex probe and 80 mm needles for the PENG block (Figure 1), as well as a high-frequency linear probe and 50 mm needles (Stimuplex, Braun, Melsungen, Germany) to perform FLCN block. Sealed and controlled batches of levobupivacaine 0.25% (Abbvie, Chicago, IL, USA) and ropivacaine 0.75% (Fresenius Kabi, Bad Homburg, Germany) were used exclusively for the present study. Ropivacaine was diluted 50% with 0.9% saline to achieve a concentration of 0.375%. A total of 20 mL of these drugs was administered to perform the PENG blocks, and 5 mL to perform the FLCN blocks.

After performing the blocks, the onset time for the establishment of analgesia was explored, considering the PENG block as effective after carefully and progressively elevating the fractured limb through passive movement until reaching 45° of flexion without observing any signs of pain in the patient.

Subsequently, spinal surgical anesthesia was administered by the same anesthesiologist in a sitting position, using in all cases 5 mg of hyperbaric bupivacaine 0.5% (Braun, Melsungen, Germany) with 10 μg of fentanyl (Kern, Madrid, Spain). The decision was made to perform spinal anesthesia over general anesthesia because, as discussed in the Introduction, although previous studies have not demonstrated differences between them regarding primary outcomes [3,4,5], some benefits in favor of spinal anesthesia have been observed in secondary outcomes like hospital stay and postoperative complications [6,7,8], facts that have tilted the preference towards the use of spinal anesthesia in routine clinical practice, particularly in elderly patients [9].

After surgery, which was approached in the lateral decubitus position, the patients were transferred to the post-anesthesia care unit and later to the Traumatology hospitalization ward when their clinical condition allowed.

Serial assessments of the intensity of pain were made using the VAS, PAINAD, and AlgoPlus scales at the following specific moments: just before performing the blocks, 10 min after performing the blocks, at the time of sitting for the spinal anesthesia, at the time of discharge from the post-anesthesia care unit, and at 6 h–12 h–24 h–48 h after block administration. The end of the follow-up of each patient took place at the moment in which the patient demanded the need for rescue analgesia, or 48 h after performing the blocks.

Analgesic rescue consisted of paracetamol 1 g IV as the first drug of choice. If pain persisted, metamizole 2 g IV (dexketoprofen 50 mg IV in case of allergy) and tramadol 100 mg IV were administered in that order, as needed based on pain control.

### 2.4. Data Collection and Outcomes

The primary, patient-centered outcome of this study is to compare the duration of analgesia (measured as the time elapsed until the need for analgesic rescue or until the end of the follow-up period, depending on the case) and analgesic quality (through the measurement of pain intensity at the moments described earlier using the three scales proposed) provided by these local anesthetics. The secondary outcomes are to compare the onset time between groups and to describe the need and type of rescue analgesics and any possible associated adverse effects, recording the occurrence of the following potential complications associated with the technique during the postoperative follow-up: postoperative nausea and/or vomiting, local anesthetic toxicity, delirium (diagnosed using the Confusion Assessment Method—CAM scale), hyperglycemia, or infection (diagnosed as the onset of fever and leukocytosis with the need for antibiotic treatment).

In the case of the VAS, the most widespread scale for pain assessment, active patient collaboration is required for its use [24,25]. Since it is common in this context to encounter patients with cognitive impairment, we found it essential to use other analgesic scales whose use is independent of the patient’s collaboration in order to ensure that an analgesic assessment was obtained without compromising the precision of its results in all cases of our study. Given that the use of these types of scales is not as widespread in research as in the case of the VAS scale, because they assess different psychometric properties, and there is no consensus on the superiority of one of them, we decided to use two of the scales validated in the Spanish language in patients with advanced dementia and communication difficulties: the PAINAD and AlgoPlus assessment scales [26,27,28]. The rationale for including patients with cognitive impairment in this study, a subgroup typically excluded from clinical trials, stems from the increasing prevalence of this subgroup due to the widespread aging of our society, particularly evident in hip fractures, as demonstrated by the literature provided in the Introduction. This study advocates for the inclusion of patients with cognitive impairment in research, promoting greater generalizability of scientific studies across the general population.

### 2.5. Statistical Analysis

Based on the pharmacokinetics of both local anesthetics and previous studies [23], we used the G*Power 3.1 software (Faul F, Erdfelder E, Lang AG, Buchner A; Heinrich-Heine-Universität, Düsseldorf, Germany) to calculate the sample size. We considered as significant a difference in block duration of at least 3 h, with a variability of 50%. The significance level, or alpha error, was set at 5%. The beta error was 20%. A total of 108 patients were obtained, assuming a 10% loss; a recruitment goal of 120 patients was set. Randomization was performed using SPSS 25.0 (IBM Corporation, New York, NY, USA), which generated two treatment groups and assigned the recruited patients to each group through simple randomization. The principal investigator of the clinical trial was responsible for generating the randomization sequence. The recruitment and assignment of patients to their corresponding treatment groups following the sequence, the execution of the blocks, and the collection of data were performed by the research team members on duty during hip fracture surgery. A descriptive analysis plan for the variables collected was approved by the authors before the analysis began. Non-categorical variables are expressed as medians (interquartile range—IQR). Categorical variables are expressed as percentages or frequencies. After conducting the appropriate normality tests (Kolmogórov–Smirnov), differences in the samples were determined using Student’s *t*-tests or Mann–Whitney U tests. The comparison analysis of the categorical variables was carried out using Chi-square tests with contingency tables. The default significance level was 5% (*p* < 0.05). Calculations were performed using SPSS version 25.0 (IBM Corporation, New York, NY, USA).

## 3. Results

**Figure 2:**
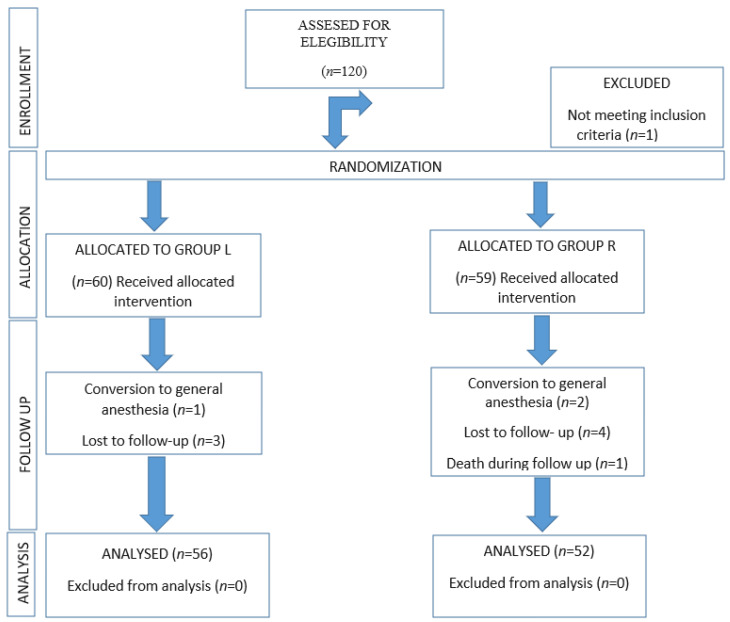
Consolidated Standards of Reporting Trials (CONSORT) flow diagram describing patients progress through the study.

A participant flow diagram is shown in Figure 2. From February 2021 to November 2021, 120 patients were assessed for eligibility. In total, 1 patient was excluded for not meeting the inclusion criteria (age < 65 years), and the remaining 119 patients were randomized. Sixty patients were enrolled in the Levobupivacaine (“L”) group, and fifty-nine patients were enrolled in the Ropivacaine (“R”) group. During follow-up, one patient was lost in group L due to conversion to general anesthesia, and three patients were lost to follow-up. In group R, two patients were lost due to conversion to general anesthesia, four of them were lost during the follow-up period, and another one died just after being discharged to the Traumatology ward due to a pulmonary embolism; therefore, these data were removed from the study as it was not possible to determine the total duration of the block in this case. The aforementioned patients were removed from the data analysis, so fifty-six patients in the L group and fifty-two patients in the R group were analyzed. Therefore, 108 patients were analyzed, thus complying with the sample calculated in the design of the trial.

Patient demographic characteristics, type of fracture and surgery, and duration of surgery were similar in the two groups, which were also consistent with those found in routine clinical practice in our setting (Table 1).

The performed combined technique and the subsequent spinal anesthesia were clinically successful in all the analyzed patients, not requiring any other supplementary intraoperative analgesia.

**Figure 3:**
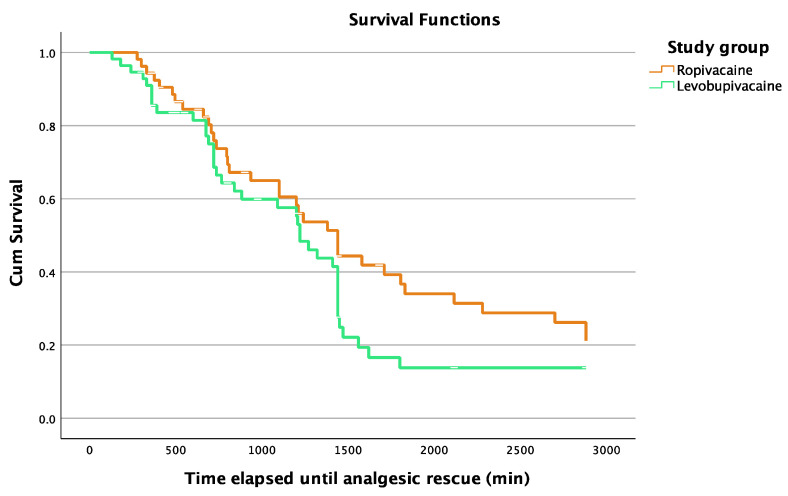
Survival curve representing the evolution through time in each group regarding the analgesic duration of the technique.

Regarding the primary outcome of this study, there was no statistically significant difference in the postoperative analgesic duration of the block between the L and R groups: 861.0 (960) min (median (IQR)) vs. 1205.0 (1379) min; *p* = 0.069). The behavior of the groups for the analgesic duration can be visually appreciated in Figure 3. In addition, there was no statistically significant difference between the groups in the assessment of pain intensity in any of the measurements made (Table 2).

With respect to the secondary outcomes, there was no statistically significant difference with respect to the onset time of the block between the L and R groups: 9.0 (4) min (median (IQR)) vs. 10.0 (6) min; *p* = 0.977). Regarding the overall analgesic rescue therapies used, 12 patients (11.1%) did not require any analgesic rescue after the 48 h follow-up. For the rest of the patients, 1 g IV paracetamol was successful as the first rescue drug in 74 of the patients (68.5%), while 15 of them required an addition of 2 g IV metamizole (13.9%), 2 required 50 mg IV dexketoprofen (1.9%), and 5 of them required an addition of more potent rescue analgesia in the form of opioid drugs (4.6%; 3 patients received 100 mg IV tramadol, 1 patient received 1 g IV paracetamol plus 100 mg tramadol, 1 patient received 2 mg subcutaneous morphine chloride).

The possible adverse effects observed in both groups during the follow-up period were as follows: 3 patients presented delirium (2.8%), and 12 patients (11.1%) suffered from postoperative infection. None of the patients experienced postoperative nausea, vomiting, hyperglycemia, or local anesthetic toxicity.

## 4. Discussion

Considering the main objective of this study, no statistically significant difference was observed in the duration of the postoperative analgesic effect (the time until the need for analgesic rescue) between levobupivacaine and ropivacaine at equipotent doses in the context of hip fracture surgery in elderly patients. This is an interesting finding since the literature, although contrasting results have been reported, strongly supports the idea that levobupivacaine generally has a longer block duration and higher potency with respect to ropivacaine [29]; hence, the authors of this study expected to find that levobupivacaine would have a longer block duration than ropivacaine. Precisely due to this, we also found a fact that deserves to be mentioned: the difference between the median duration of both groups is 344 min in favor of group R. This difference is intriguing enough to suggest its clinical relevance in the context of routine clinical practice as it extends the duration of the indisputable benefits associated with effective pain control in elderly patients during the postoperative period by 5.73 h. If statistically proven with the conduct of future studies with greater statistical power, this difference would be of great utility in daily clinical practice.

In Figure 2, this fact can be observed visually: group L experienced a marked end of analgesia between 1200 and 1500 min, such that at 1500 min, around 20% of the patients remained under analgesia. However, the end of analgesia in group R occurs more progressively, so that at 1500 min, more than 40% of the patients remained under analgesia. Globally, we can observe that after 1500 min, the slope of the survival curve lessens in both groups, and at the end of the 48 h follow-up period (2800 min), a greater number of patients remained under analgesia in group R.

One reason to explain the consistently longer duration of levobupivacaine compared to ropivacaine in the previous literature and why this has not been the case in our study is that in the majority of previous studies, these local anesthetics were used at the same concentration [17]. Additionally, it has been described in the literature that the potency of levobupivacaine is generally 50% higher than that of ropivacaine in suppressing tetrodotoxin-resistant sodium ion channels [30]. This could be explained by the greater lipid solubility and increased protein binding of levobupivacaine, as well as the different osmolality between the presentations of the two drugs, since the concentration of levobupivacaine is denoted on the drug label as the concentration of the base of the molecule, while the concentration of ropivacaine is presented as the hydrochloride salt [29,31]. In this way, it makes sense that when comparing equal concentrations of these two local anesthetics, levobupivacaine exhibits a longer duration than ropivacaine. However, due to these pharmacokinetic differences, we decided to use equipotent concentrations of these drugs in this study to account for the higher relative potency of levobupivacaine, noting that under these conditions, the difference between the two local anesthetics dissipates, as it was showed that the onset and duration of nerve block induced by equimolar doses of 2 LAs were similar on isolated nerves [32]. In Kim et al.’s study [33], where the same equipotent doses of levobupivacaine and ropivacaine were used as in our study, no difference between them was observed concerning the investigated outcomes, except for a shorter onset time in favor of ropivacaine.

Furthermore, regarding the analgesic quality provided by the studied anesthetics, no statistically significant differences were observed between groups in any of the pain scores obtained for each temporal assessment using the three employed scales, as depicted in Table 2. In the study by Borghi et al. [34], it was observed that the analgesic quality of 0.25% levobupivacaine was comparable to that provided by an equipotent concentration of 0.4% ropivacaine. However, the anesthesia produced by levobupivacaine was superior to that provided by ropivacaine when used at equivalent concentrations of 0.25% in a similar clinical scenario, a fact that also supports the previously discussed considerations regarding the different relative potencies between the two drugs.

Regarding the secondary outcomes, there was no statistically significant difference between groups in the onset time, as we expected based on the previous literature [17]. The technique offers a quick onset, which is highly appreciated. In addition, after the onset of the block, a reduction of around 50% in the pain scores is observed in both groups according to the three scales used, reaching low pain score levels, which reduces the patient’s discomfort at the time of patient transfer and mobilization for spinal anesthesia. The technique presents an attractive mean postoperative analgesic duration in both treatment groups, and first-step analgesic drugs were effective as a rescue in 84.3% of patients, having to resort to opioids as a rescue in only 4.6% of cases. The low incidence of adverse effects that occurred in our study makes this a recommendable block to be used in clinical practice.

It is important to emphasize that this study includes in its sample patients with cognitive impairment without compromising the precision of its results, thanks to the use of specific scales tailored for these patients. This subgroup is typically excluded from clinical trials, despite being a subgroup increasingly prevalent due to the widespread aging of our society, and this group is particularly common in hip fractures. An example of this is the fact that in around 25% of the patients in our study, it was not possible to assess pain using the VAS due to lack of patient cooperation, attributed to the presence of cognitive impairment, as can be observed in Table 2 regarding the lower number of patients analyzed with the VAS in comparison to the PAINAD and AlgoPlus scales. On the other hand, given the similar pain score obtained between the three scales, this suggests the usefulness of pain assessment scales that are independent of the patient’s cognitive cooperation in the context of clinical trials. As such, these scales may contribute to ensuring the inclusion of patients with cognitive impairment in research, promoting a more thorough generalization of scientific advancements.

## 5. Limitations

To the best of our knowledge, this study is the first to compare the duration and quality of analgesia provided by these local anesthetics in equipotent doses in the context of the PENG block or other fascial blocks. This is why the equipotence relationship chosen for this work is based on the results of studies carried out in the context of neuraxial and plexus blocks, which were executed over mixed sensory–motor nerve structures of a large caliber and covered by a thick myelin sheath. Extrapolating the equipotence relationship defined in these studies could be questionable as the PENG block is performed on small terminal sensory nerve fibers based on the ultrasound localization of the compartments through which these fibers run. This is because it has been described that clinical circumstances such as the type of block, the block technique, and site of deposition of local anesthetics, and even the magnitude of surgery, can largely affect their behavior during routine clinical practice given its complexity and variability [23,31] beyond the mere pharmacokinetic factors derived from classical studies on the potency of local anesthetics. So, it is necessary to carry out new studies that establish an equipotence relationship and compare the duration and analgesic quality between these local anesthetics at the level of fascial blocks. Furthermore, it would be interesting to conduct studies with greater statistical power, with the aim of being able to assess if it would be possible to statistically demonstrate the difference between median durations of block observed in the present study in favor of ropivacaine given that, in our case, due to the non-normality of the distribution of this variable, the Mann–Whitney U test was used to test the hypotheses which, being a non-parametric test, makes it more difficult to demonstrate statistically significant differences.

The performance of the blocks and pain intensity assessments was assessed by different anesthesiologists, even though the entire team was familiar with the technique and with the use of the scales.

Although a 48 h follow-up seemed sufficient given the typical duration of locoregional techniques, it would have been necessary to extend the follow-up period to more precisely define the total duration of both anesthetics since at 48 h follow-up, 11% of the patients still did not need rescue analgesics.

Due to the high patient care burden in our center, we were unable to allocate the necessary human resources to implement proper blinding of the intervention. Nevertheless, in our opinion, this is not a factor compromising objectivity in the measurement of variables, as the determination of the main outcomes relied primarily on assessments made by the patients themselves without the intervention of the research staff, and the patients were unaware of the specific local anesthetic administered. The analgesic duration depended on the moment in which the patient requested rescue analgesia, and the pain score was likewise reported by the patients in the case of the VAS scale, or objectively determined through the rigorous application of the Painad and AlgoPlus scales.

## 6. Conclusions

There are no statistically significant differences in terms of block duration, analgesic quality, or onset time between the groups studied, which implies that levobupivacaine and ropivacaine share a similar clinical profile when used in the PENG block at an equipotent concentration. New, more powerful studies on this topic would be necessary to thoroughly explore the interesting and unexpected findings described in relation to the longer median duration of ropivacaine compared to levobupivacaine.

## Figures and Tables

**Table 1:** Patient demographic and clinical parameters.

	Group L (*n* = 56)	Group R (*n* = 52)
Age, years	88.0 (13.0)	87.0 (7)
Gender, *n* (%)		
Male	15 (26.8)	9 (17.3)
Female	41 (73.2)	43 (82.7)
Height, cm	1.56 (0.13)	1.57 (0.11)
Weight, kg	65.5 (16.0)	61.5 (15.75)
Body Mass Index, kg/m^2^	25.14 (4.86)	24.36 (5.65)
Duration of surgery, min	49.0 (25.0)	50.0 (19.0)
Type of fracture, *n* (%)		
Sub-capital	24 (42.9)	14 (26.9)
Pertrochanteric	31 (55.4)	36 (69.2)
Sub-pertrochanteric	1 (1.8)	0 (0)
Others		2 (3.8)
Type of surgery, *n* (%)		
Partial Hip Arthroplasty	24 (42.9)	14 (26.9)
Endomedullary Nailing	32 (57.1)	37 (71.2)
Others	0 (0)	1 (1.9)

Data are presented as median (IQR) or number (percentage). Group L: levobupivacaine, Group R: ropivacaine.

**Table 2:** Pain intensity scores measured by VAS, PAINAD, and AlgoPlus scales.

	Group L	Group R	*p*
Arrival to OT			
VAS R	0.0 (3) (*n* = 38)	0.00 (4) (*n* = 40)	0.674
VAS A	8.0 (5) (*n* = 37)	8.00 (4) (*n* = 39)	0.487
PAINAD R	0.0 (0) (*n* = 56)	0.00 (0) (*n* = 52)	0.273
PAINAD A	6.0 (4) (*n* = 56)	5.00 (5) (*n* = 52)	0.239
AlgoPlus R	0.0 (0) (*n* = 56)	0.00 (0) (*n* = 52)	0.431
AlgoPlus A	4.0 (2) (*n* = 56)	3.00 (2) (*n* = 52)	0.872
10 min after block			
VAS R	0.00 (0) (*n* = 38)	0.00 (0) (*n* = 40)	0.479
VAS A	3.00 (4) (*n* = 38)	2.00 (4) (*n* = 39)	0.346
PAINAD R	0.00 (0) (*n* = 56)	0.00 (0) (*n* = 52)	0.919
PAINAD A	2.00 (2) (*n* = 56)	1.00 (3) (*n* = 52)	0.381
AlgoPlus R	0.00 (0) (*n* = 56)	0.00 (0) (*n* = 52)	0.757
AlgoPlus A	1.00 (1) (*n* = 56)	1.00 (2) (*n* = 52)	0.426
Sitting for the SAB			
VAS	0.00 (3) (*n* = 39)	0.00 (1) (*n* = 40)	0.581
PAINAD	0.00 (2) (*n* = 56)	0.00 (1) (*n* = 52)	0.443
AlgoPlus	0.00 (1) (*n* = 54)	0.00 (1) (*n* = 51)	0.294
At discharge of PCU			
VAS R	0.00 (0) (*n* = 39)	0.00 (0) (*n* = 41)	0.492
VAS A	0.00 (3) (*n* = 37)	0.00 (2) (*n* = 41)	0.481
PAINAD R	0.00 (0) (*n* = 55)	0.00 (0) (*n* = 52)	0.529
PAINAD A	0.00 (2) (*n* = 55)	0.00 (1) (*n* = 52)	0.236
AlgoPlus R	0.00 (0) (*n* = 53)	0.00 (0) (*n* = 52)	0.956
AlgoPlus A	0.00 (1) (*n* = 55)	0.00 (1) (*n* = 52)	0.248
6 h after block			
VAS R	0.00 (0) (*n* = 40)	0.00 (0) (*n* = 40)	0.913
VAS A	1.00 (4) (*n* = 38)	0.00 (3) (*n* = 40)	0.479
PAINAD R	0.00 (0) (*n* = 54)	0.00 (0) (*n* = 51)	0.364
PAINAD A	1.00 (3) (*n* = 54)	0.00 (1) (*n* = 51)	0.264
AlgoPlus R	0.00 (0) (*n* = 52)	0.00 (0) (*n* = 51)	0.093
AlgoPlus A	1.00 (2) (*n* = 54)	0.00 (1) (*n* = 51)	0.458
12 h after block			
VAS R	0.00 (0) (*n* = 29)	0.00 (0) (*n* = 34)	0.508
VAS A	2.50 (4) (*n* = 26)	2.00 (6) (*n* = 33)	1.000
PAINAD R	0.00 (0) (*n* = 43)	0.00 (0) (*n* = 45)	0.314
PAINAD A	2.00 (3) (*n* = 43)	1.00 (3) (*n* = 45)	0.539
AlgoPlus R	0.00 (0) (*n* = 41)	0.00 (0) (*n* = 44)	0.188
AlgoPlus A	1.00 (2) (*n* = 43)	1.00 (3) (*n* = 45)	0.836
24 h after block			
VAS R	0.00 (1) (*n* = 18)	0.00 (1) (*n* = 21)	0.878
VAS A	4.50 (4) (*n* = 15)	3.50 (5) (*n* = 20)	0.382
PAINAD R	0.00 (0) (*n* = 25)	0.00 (0) (*n* = 33)	0.397
PAINAD A	4.00 (2) (*n* = 25)	2.00 (5) (*n* = 33)	0.374
AlgoPlus R	0.00 (0) (*n* = 25)	0.00 (0) (*n* = 33)	0.401
AlgoPlus A	2.50 (2) (*n* = 25)	1.50 (3) (*n* = 33)	0.174
48 h after block or at time of analgesic rescue			
VAS R	0.00 (2) (*n* = 38)	0.00 (2) (*n* = 40)	0.526
VAS A	5.00 (4) (*n* = 36)	5.00 (4) (*n* = 39)	0.864
PAINAD R	0.00 (0) (*n* = 56)	0.00 (0) (*n* = 52)	0.950
PAINAD A	3.00 (2) (*n* = 56)	3.00 (4) (*n* = 52)	0.551
AlgoPlus R	0.00 (0) (*n* = 56)	0.00 (0) (*n* = 52)	0.865
Algoplus A	2.00 (2) (*n* = 56)	2.00 (2) (*n* = 52)	0.724

Data are presented as median (interquartile range). Group L: levobupivacaine, Group R: ropivacaine, R: rest, A: activity (passive leg raising), OT: operating theatre, SAB: subarachnoid block, PCU: postoperative care unit, VAS: visual analogue scale, Painad: pain assessment in advanced dementia scale, AlgoPlus: AlgoPlus scale.

## Data Availability

Data are contained within the article.
